# Bacterial mediated green synthesis of silver nanoparticles and their antibacterial and antifungal activities against drug-resistant pathogens

**DOI:** 10.1098/rsos.230796

**Published:** 2023-10-25

**Authors:** Md. Amdadul Huq, Azmat Ali Khan, Jamilah M. Alshehri, Md. Shahedur Rahman, Sri Renukadevi Balusamy, Shahina Akter

**Affiliations:** ^1^ Department of Food and Nutrition, College of Biotechnology and Natural Resource, Chung-Ang University, Anseong, Gyeonggi-do 17546, Republic of Korea; ^2^ Pharmaceutical Biotechnology Laboratory, Department of Pharmaceutical Chemistry, College of Pharmacy, King Saud University, Riyadh 11451, Saudi Arabia; ^3^ Department of Genetic Engineering and Biotechnology, Jashore University of Science and Technology, Jashore 7408, Bangladesh; ^4^ Department of Food Science and Technology, Sejong University, Seoul 143-747, Republic of Korea; ^5^ Department of Food Science and Biotechnology, Gachon University, Seongnam 461-701, Republic of Korea

**Keywords:** green synthesis, novel silver nanoparticles, *Paenibacillus* sp. MAHUQ-63, antimicrobial activity, *Salmonella* Enteritidis, *Candida albicans*

## Abstract

In the healthcare sector, the production of bioactive silver nanoparticles (AgNPs) with antimicrobial properties is of great importance. In this study, a novel bacterial strain, *Paenibacillus* sp. MAHUQ-63, was identified as a potential candidate for facile and rapid biosynthesis of AgNPs. The synthesized AgNPs were used to control the growth of human pathogens, *Salmonella* Enteritidis and *Candida albicans*. The bacterial culture supernatant was used to synthesize the nanoparticles (NPs). Field emission transmission electron microscope examination showed spherical-shaped NPs with 15–55 nm in size. Fourier transform-infrared analysis identified various functional groups. The synthesized AgNPs demonstrated remarkable activity against *S.* Enteritidis and *C. albicans*. The zones of inhibition for 100 µl (0.5 mg ml^−1^) of AgNPs against *S.* Enteritidis and *C. albicans* were 18.0 ± 1.0 and 19.5 ± 1.3 mm, respectively. The minimum inhibitory concentrations were 25.0 and 12.5 µg ml^−1^ against *S.* Enteritidis and *C. albicans*, respectively. Additionally, the minimum bactericidal concentrations were 25.0 µg ml^−1^ against both pathogenic microbes. The field emission scanning electron microscopy analysis showed that the treatment of AgNPs caused morphological and structural damage to both *S.* Enteritidis and *C. albicans*. Therefore, these AgNPs can be used as a new and effective antimicrobial agent.

## 1. Introduction

Nanobiotechnology is a rapidly expanding field in biological sciences that has various applications in the health sector, particularly for the production of bioactive nanomaterials such as nanoparticles (NPs) and nanoconjugates for controlling several diseases. Green nanotechnology-based NPs and nanoconjugates have attracted huge interest because of their extensive biomedical applications [1–3]. NPs have shown promising results as alternative drugs for human diseases, including cancer, diabetes, wound healing and infectious diseases [4–9]. Various physical, chemical and biological methods are commonly used for synthesizing different types of NPs [10,11]. Among different chemical and physical methods, chemical reduction, electrochemical, physiochemical and microwave irradiation are usually used for the production of NPs [11]. The synthesis of NPs using macro- and micro-organisms, such as extracts of plant parts, bacteria, algae and fungi, is known as biosynthesis of NPs [11–13]. The biological method is considered a safe and cost-effective method and does not have any toxic effects on the environment. By contrast, chemical and physical approaches have various hazardous effects on the environment due to the use of toxic chemicals and the production of toxic byproducts [14]. Biosynthesized NPs have several advantages, including less toxicity and high stability [11,15,16]. Moreover, the biosynthesized NPs show a wide range of biomedical applications such as antioxidant, antimicrobial, anti-inflammation and anti-cancer agents as well as promising carriers for various drug delivery systems [1–5].

Among various NPs, biosynthesized silver nanoparticles (AgNPs) have gained significant attention from researchers due to their numerous applications, particularly in the medical field [17,18]. Microbe-mediated synthesis of bioactive AgNPs is a facile and eco-friendly approach [11]. Among different microorganisms, bacteria have gained more attention from researchers for the synthesis of AgNPs due to their ease of handling and manipulation, which make them perfect for the large-scale synthesis of NPs. Bacteria secrete numerous bioactive compounds in the culture supernatant, including enzymes, proteins, hormones, ions, polysaccharides and pigments, which play an important role during NP synthesis [11,19]. Nicotinamide adenine dinucleotide (NADH)-dependent reductases, as well as sulfur-containing proteins, play an essential role in the reduction and stabilization of NPs [19–21]. Several bacteria have been reported for the biosynthesis of AgNPs, including *Microvirga rosea* [22], *Bacillus sonorensis* [23], *Terrabacter humi* [24], etc. There are many recent reports regarding the potent antimicrobial effect of biosynthesized AgNPs against human pathogens [25–27]. Due to the strong antimicrobial action of AgNPs, they are used in the development of footwear, cosmetics, wound dressings and other medical devices [19].

The emergence of multi-drug-resistant (MDR) microorganisms poses a severe threat to global public health [28]. With the growing resistance of various pathogenic microorganisms to conventional antibiotics, there is a critical need for novel and effective antimicrobial agents. *Salmonella* Enteritidis causes serious foodborne illness in humans [29,30]. Previous studies have reported the drug resistance of *S.* Enteritidis [30]. Candidiasis, a fungal infection commonly caused by *Candida albicans*, poses a significant challenge to human health due to the emergence of drug-resistant strains [31,32]. *Candida albicans* is responsible for a wide range of infections in humans, such as genital yeast infections, oral infections and skin infections, etc.

In the present study, a novel bacterial strain *Paenibacillus* sp. MAHUQ-63 was isolated from a pumpkin garden and employed for the eco-friendly synthesis of bioactive AgNPs. The synthesized AgNPs were characterized using various instruments such as UV–visible (UV–Vis) spectrophotometer, field emission transmission electron microscope (FE-TEM), X-ray diffraction (XRD), dynamic light scattering (DLS), Fourier transform-infrared (FTIR), etc. Furthermore, the AgNPs were evaluated for their antibacterial and antifungal activity against *S.* Enteritidis and *C. albicans*.

## 2. Material and methods

### 2.1. Materials

The pathogenic strains *S.* Enteritidis [ATCC 13076] and *C. albicans* [KACC 30071] were obtained from ATCC and KACC, respectively. All standard antibiotics discs were bought from Oxoid Ltd, England.

### 2.2. Isolation and molecular identification

In this study, a bacterial strain capable of producing AgNPs was isolated from soil sample of a pumpkin garden in Anseong, South Korea. The isolation process involved serial dilution of the soil sample in sterile NaCl solution (0.8%), plating the dilutions onto R2A agar plates and incubating them at 30°C for 72 h. Colonies were then cultured in R2A broth and treated with AgNO_3_ solution for 48 h at 30°C. The strain MAHUQ-63 which showed strong reduction efficacy was selected for further analysis and identified as *Paenibacillus* sp. MAHUQ-63 by constructing a phylogenetic tree using the MEGA6 program and neighbour-joining algorithm based on the 16S rRNA gene sequence [33–35].

### 2.3. Cultural, physiological and biochemical characterization of strain MAHUQ-63

The growth characteristics of strain MAHUQ-63 were studied using various methods. To determine the optimal growth conditions, strain MAHUQ-63 was grown on several agar media at 30°C for 3 days. The optimal growth temperature was determined by incubating strain MAHUQ-63 on R2A agar at different temperatures. The optimal pH for growth was determined using R2A broth medium. Cell morphology, including shape and size, was examined using transmission electron microscopy (TEM). The strain's oxidase, catalase, urease and DNase activities, as well as its ability to hydrolyse starch, casein, gelatine and Tween 80, were evaluated using the methods described by Huq *et al.* [36]. The API kits (bioMérieux) were used to assess additional physiological and biochemical characteristics of strain MAHUQ-63, following the manufacturer's instructions.

### 2.4. Green synthesis of silver nanoparticles

The strain MAHUQ-63 was cultured in R2A broth medium (100 ml) with shaking (180 r.p.m.) for 3 days at 30°C. AgNPs were synthesized by adding silver nitrate solution (final concentration 1.5 mM) to the culture supernatant. The reaction mixture was kept in the dark at 33°C with constant agitation (180 r.p.m.) for 2 days. The synthesis of AgNPs was monitored by observing colour changes and UV–Vis spectrophotometer analysis. The synthesized AgNPs were collected through centrifugation and washed with deionized water [13].

### 2.5. Characterization of synthesized silver nanoparticles

UV–Vis spectrophotometer was used to assess the kinetic behaviour of the synthesized AgNPs. The biosynthesized AgNPs were scanned from 300 to 800 nm to determine their absorbance. The morphology, size, purity, distribution and elemental composition of the biosynthesized NPs were analysed using FE-TEM. FE-TEM imaging was performed on air-dried biosynthesized AgNPs suspension on a grid. XRD analysis was carried out to determine the crystallinity of the synthesized AgNPs using CuKα radiation. The XRD analysis was carried out using a diffractometer in the range of 30–80° (2*θ*). The surface chemistry of the synthesized AgNPs was checked by FTIR spectroscopy. Additionally, the hydrodynamic diameters and polydispersity index of *Paenibacillus* sp. MAHUQ-63-mediated synthesized AgNPs were determined using DLS with deionized water as the dispersal medium. The DLS analysis was performed at 25°C with a scattering angle of 12° using Malvern Zetasizer Nano ZS90.

### 2.6. Antimicrobial activity

The antimicrobial potential of biosynthesized AgNPs was examined against two pathogens, *S.* Enteritidis and *C. albicans*, by the disc diffusion method [37,38]. Briefly, both pathogens were cultured overnight in Mueller–Hinton (MH) broth medium and 100 µl of each culture was spread on MH agar plates. A solution of 1 mg of biosynthesized AgNPs in 2 ml of autoclaved distilled water was prepared. Paper discs were soaked with 50 and 100 µl of AgNPs solution, and placed on the surface of the agar plates. To compare the effectiveness of the AgNPs, six different antibiotics, namely novobiocin (30 µg disc^−1^), penicillin G (10 µg disc^−1^), erythromycin (15 µg disc^−1^), oleandomycin (15 µg disc^−1^), vancomycin (30 µg disc^−1^) and lincomycin (15 µg disc^−1^), were also tested against *S.* Enteritidis and *C. albicans* as controls. The MH agar plates were then incubated at 37°C for 24 h, and the zone of inhibition (ZOI) was measured in mm [39,40].

### 2.7. Minimum inhibitory concentration and minimum bactericidal concentration

The minimum inhibitory concentration (MIC) and minimum bactericidal concentration (MBC) were evaluated for both pathogenic strains, *S.* Enteritidis and *C. albicans*. The MIC of AgNPs was determined using broth microdilution assay in 96-well ELISA plates. The pathogenic strains were cultured in MH broth, and the AgNPs concentrations ranged from 1.56 to 100 µg ml^−1^. The lowest concentration of AgNPs that inhibited microbial growth was recorded as the MIC. MBC was identified by streaking 10 µl of each set from the 96-well plates onto MH agar plates and incubating at 37°C for 24 h. The AgNP concentration at which no visible bacterial or fungal growth was observed was considered as the MBC. The antimicrobial activity of biosynthesized AgNPs was also determined using the disc diffusion method, where the ZOI was measured. Six different antibiotics were used as controls against *S.* Enteritidis and *C. albicans*. The experimental methods followed previous studies [37,39,41,42].

### 2.8. Morphological evaluation

To observe the antibacterial and antifungal mechanisms of biosynthesized AgNPs against *S.* Enteritidis and *C. albicans*, the cells of both pathogens were treated with and without biosynthesized AgNPs at MIC and observed under field emission scanning electron microscopy (FE-SEM). The cells of both pathogens were cultured overnight in MH broth and adjusted to a concentration of 1 × 10^7^ colony-forming units (CFU) ml^−1^. The cells were washed using a buffer and fixed by glutaraldehyde (2.5%) and osmium tetroxide (1%). The fixed cells were dehydrated using ethanol at different concentrations and dried using a desiccator. The morphological and structural changes of *S.* Enteritidis and *C. albicans* were observed by FE-SEM [40,43].

## 3. Results

### 3.1. Molecular identification of silver nanoparticles producing bacteria

**Figure 1.**
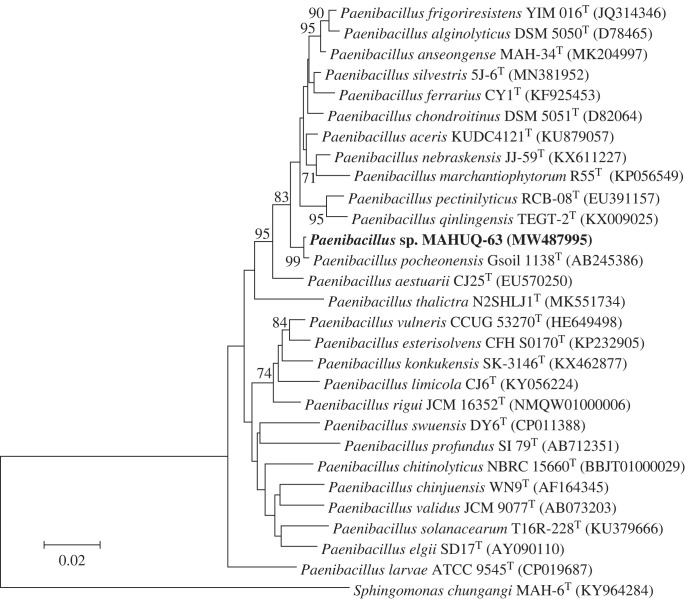
Phylogenetic tree of the isolated strain *Paenibacillus* sp. MAHUQ-63.

The 16S rRNA gene sequencing was conducted on strain MAHUQ-63, which resulted in a 1465 bp sequence. The sequence was submitted to the GenBank/EMBL/DDBJ database with an accession number MW487995. The 16S rRNA gene sequence revealed that strain MAHUQ-63 showed high similarity (98.7%) to *Paenibacillus pocheonensis* Gsoil 1138^T^. The phylogenetic analysis assured the close relationship between the strain MAHUQ-63 and the members of the genus *Paenibacillus* and formed a monophyletic clade with *P. pocheonensis* Gsoil 1138^T^ (figure 1). The deposited accession number of strain MAHUQ-63 is KACC 22244.

### 3.2. Cultural, physiological and biochemical characterization of strain MAHUQ-63

**Figure 2.**
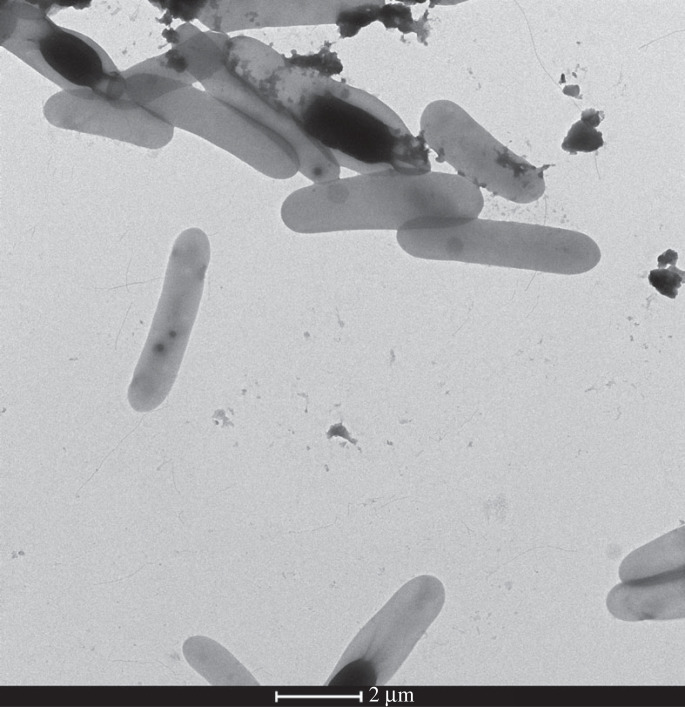
Cells of the isolated strain *Paenibacillus* sp. MAHUQ-63.

The morphology and biochemical characteristics of strain MAHUQ-63 were investigated. The cells of strain MAHUQ-63 were observed to be rod-shaped, as shown in figure 2. On R2A agar medium, the colonies of strain MAHUQ-63 grew well from 28 to 30°C temperature at pH 7.0. Strain MAHUQ-63 exhibited positive results for both catalase and oxidase activities. The strain showed weak activity for the hydrolysis of urea and gelatine. The ability of strain MAHUQ-63 to reduce nitrate to nitrite was observed, but it was unable to ferment glucose. Strain MAHUQ-63 demonstrated various enzyme activities, including positive for lipase (C14), α-galactosidase and β-galactosidase, esterase lipase (C8), napthol-AS-BI-phosphohydrolase, acid phosphatase, β-glucuronidase and α-fucosidase, and negative for esterase (C4), leucine arylamidase, trypsin and α-mannosidase, etc. Strain MAHUQ-63 can assimilate d-glucose, d-maltose, d-mannose, gluconate, d-mannitol, triosodium citrate and malic acid as a source of carbon.

### 3.3. Biosynthesis of silver nanoparticles

**Figure 3.**
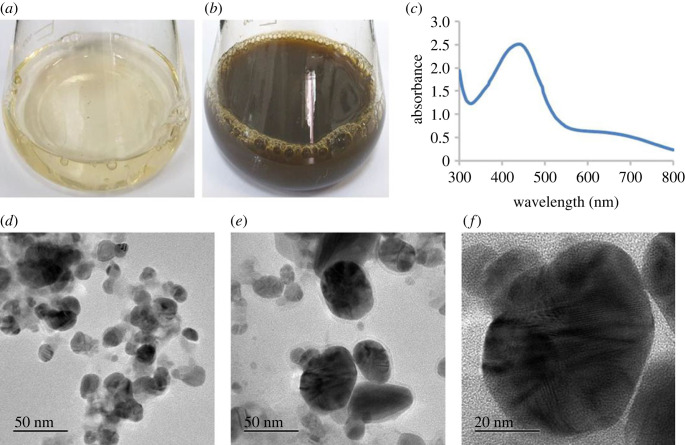
Control (AgNO_3_ in R2A broth) (*a*), biosynthesized AgNPs (*b*), absorption peak (*c*) and FE-TEM analysis of *Paenibacillus* sp. MAHUQ-63-mediated synthesized AgNPs (*d–f*).

The biosynthesis of AgNPs was indicated by the sequential colour change of the bacterial culture supernatant and AgNO_3_ mixture from watery yellow to dark brown during the incubation period (figure 3*a*,*b*) [26,44]. In this study, the extracellular method was used, which is a cost-effective, eco-friendly and easy approach compared with physical and chemical methods [45,46]. It is well-documented that microbial cells secrete various biomolecules such as amino acids, proteins, enzymes, pigments, flavonoids and carbohydrates for the biosynthesis of NPs [11,47]. The reductase enzymes secreted by microorganisms play a vital role in the synthesis of metal NPs through the reduction of metal ions [11,48,49]. Thus, the culture supernatant of *Paenibacillus* sp. MAHUQ-63 has the potential to reduce Ag^+^ ions to AgNPs, which was confirmed by the colour change observed in this study.

### 3.4. Characterization of synthesized silver nanoparticles

The confirmation of AgNP formation was made using UV–Vis absorbance spectra, which showed a specific peak at 440 nm (figure 3*c*) [24]. This specific peak was sharper and narrower than previously reported in other studies [13,50], suggesting high-quality AgNP formation [51].

TEM images of the biosynthesized AgNPs using *Paenibacillus* sp. MAHUQ-63 culture supernatant showed that the particles were spherical in shape and ranged in size from 15 to 55 nm. Moreover, the synthesized AgNPs were uniformly distributed without agglomeration, indicating their good stability (figure 3*d–f*). Previous reports have also shown the biosynthesis of AgNPs using bacteria such as *Arthrobacter bangladeshi*, *Pseudomonas* sp. and *B. sonorensis* with varying sizes ranging from 12 to 50, 10 to 40 and 13 to 50 nm, respectively [19,23,38].

**Figure 4.**
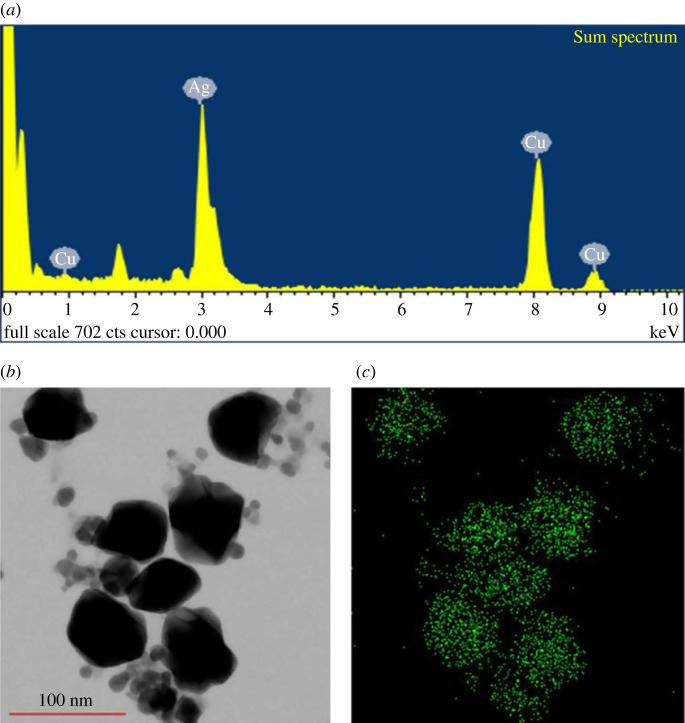
EDX spectrum (*a*), FE-TEM image (*b*) and distribution of silver element in sample (*c*).

The synthesized nanomaterial was characterized by energy dispersive X-ray (EDX) to determine its elemental composition and distribution. The results showed that silver was the major element in the nanomaterial (figure 4*a–c*). The characteristic peak of silver was observed at 3 keV in the EDX spectrum (figure 4*a*). Table 1 summarizes the elemental percentages obtained by EDX analysis.

**Table 1.** Chemical elements present in *Paenibacillus* sp. MAHUQ-63-mediated green synthesized AgNPs.

element	weight%	atomic%
Cu K	33.16	45.72
Ag L	66.84	54.28
totals	100.00	100.00

**Figure 5.**
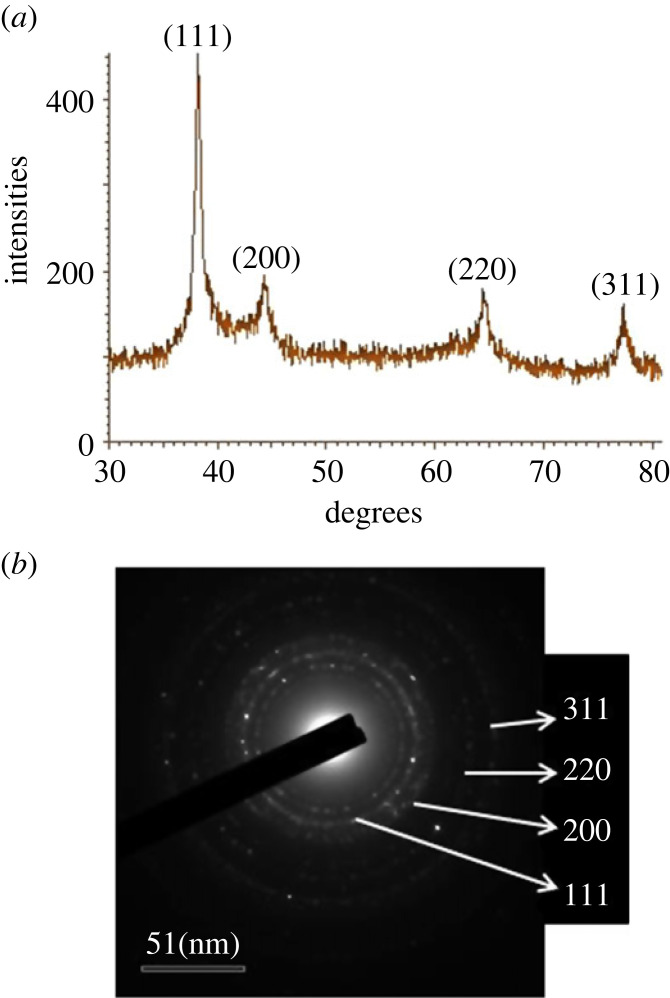
XRD spectrum (*a*) and selected area electron diffraction (SAED) pattern (*b*) of *Paenibacillus* sp. MAHUQ-63-mediated green AgNPs.

The XRD analysis revealed four peaks at 38.50°, 44.43°, 64.60° and 77.82° 2*θ* values, which matched with the face-centred cubic crystal system of AgNPs (figure 5*a*). The XRD spectrum confirmed the crystallinity of the biosynthesized AgNPs. These results are in agreement with previous studies of AgNPs biosynthesized by plants and microbes [13,52]. The selected area electron diffraction (SAED) pattern also verified the crystallinity of AgNPs, and the inner ring in the SAED pattern corresponded to the (111) plane, which is a typical diffraction ring of AgNPs (figure 5*b*). The SAED pattern results were consistent with the XRD analysis results [53].

**Figure 6.**
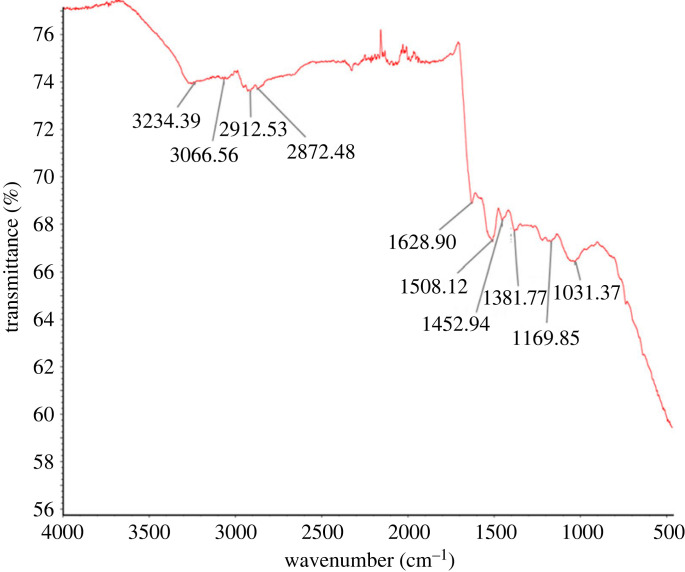
FTIR spectra of AgNPs synthesized by *Paenibacillus* sp. MAHUQ-63.

The FTIR spectrum of biosynthesized AgNPs was investigated, and the results are shown in figure 6. The FTIR bands at 3234.39 and 3066.56 cm^−1^ were assigned to the O–H stretching of alcohols and/or N–H of primary amines. The FTIR bands at 2912.53 and 2872.48 cm^–1^ corresponded to the C–H stretching of alkane. FTIR bands at 1628.90, 1508.12, 1452.94, 1381.77, 1169.85 and 1031.37 cm^–1^ indicated C=C (olefin), N–O (nitro compound), methyl group C–H (alkane), O–H (phenol) group, C–O (alkyl aryl ether) group and S=O (sulfoxide) group, respectively. These various functional groups suggested the participation of different biomolecules in the reduction and stabilization of AgNPs. The results are consistent with previous studies that reported the existence of various biomolecules in cell extracts that can reduce Ag^+^ ions and stabilize NPs [23,50]. Therefore, the FTIR analysis supports the hypothesis that the biosynthesized AgNPs are stabilized by biomolecules, which is in line with the findings of previous studies [23,50].

**Figure 7.**
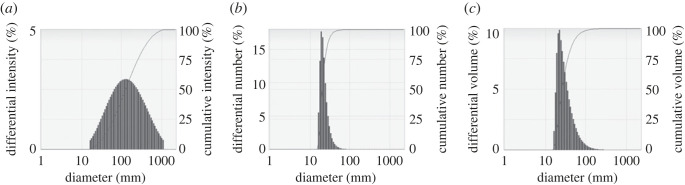
DLS analysis according to intensity (*a*), number (*b*) and volume (*c*).

The hydrodynamic diameter of *Paenibacillus* sp. MAHUQ-63-mediated biosynthesized AgNPs in an aqueous system was determined to be 91.1 nm with a polydispersity index of 0.458 (figure 7). The observed polydisperse standard indicates that the synthesized AgNPs have a size distribution with varying particle sizes. The larger size of the NPs observed in DLS analysis compared with the size calculated from FE-TEM is due to the presence of water. Previous studies also reported larger hydrodynamic diameters compared with TEM-based measurements [45].

### 3.5. Antimicrobial activity

**Figure 8.**
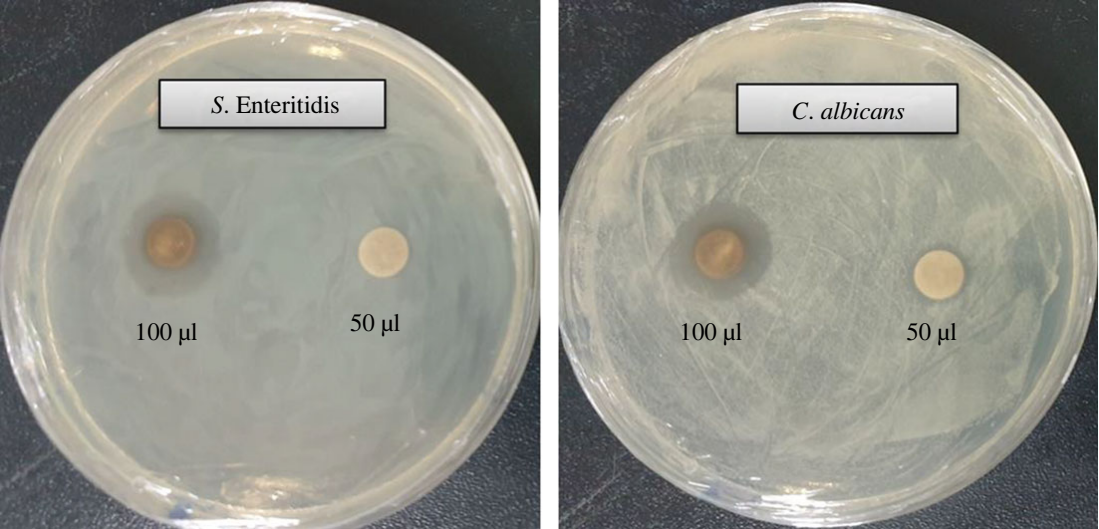
Antibacterial and antifungal effectiveness of synthesized AgNPs. Zones of inhibition against *S.* Enteritidis and *C. albicans* (50 µl and 100 µl at 500 ppm concentrations).

The emergence of drug-resistant microorganisms poses a serious threat to public health. The limitations of available antibiotics in treating infectious diseases have led to an increase in resistant microorganisms, emphasizing the urgent need for new and effective antimicrobial agents. Biosynthesized AgNPs could be strong antimicrobial agents that possess a lethal effect against pathogenic microbes. The current study investigated the antibacterial and antifungal activities of AgNPs synthesized by *Paenibacillus* sp. MAHUQ-63 against pathogenic *S.* Enteritidis and *C. albicans*. The biosynthesized AgNPs exhibited a significant inhibitory effect against both bacterial and fungal pathogens. Figure 8 depicts the clear ZOI. The ZOIs against *S.* Enteritidis and *C. albicans* were 18.0 ± 1.0 mm and 19.5 ± 1.3 mm, respectively, when treated with a 100 µl AgNPs solution at a 500 ppm concentration (table 2). Our findings suggest that biosynthesized AgNPs can effectively control pathogenic *S.* Enteritidis and *C. albicans*.

**Table 2.** Antibacterial impact of *Paenibacillus* sp. MAHUQ-63-mediated green synthesized AgNPs against *S.* Enteritidis and *C. albicans*.

pathogenic species	ZOI (mm)	
	50 µl	100 µl
*Salmonella* Enteritidis [ATCC 13076]	—	18.0 ± 1.0
*Candida albicans* [KACC 30071]	10.5 ± 1.2	19.5 ± 1.3

**Figure 9.**
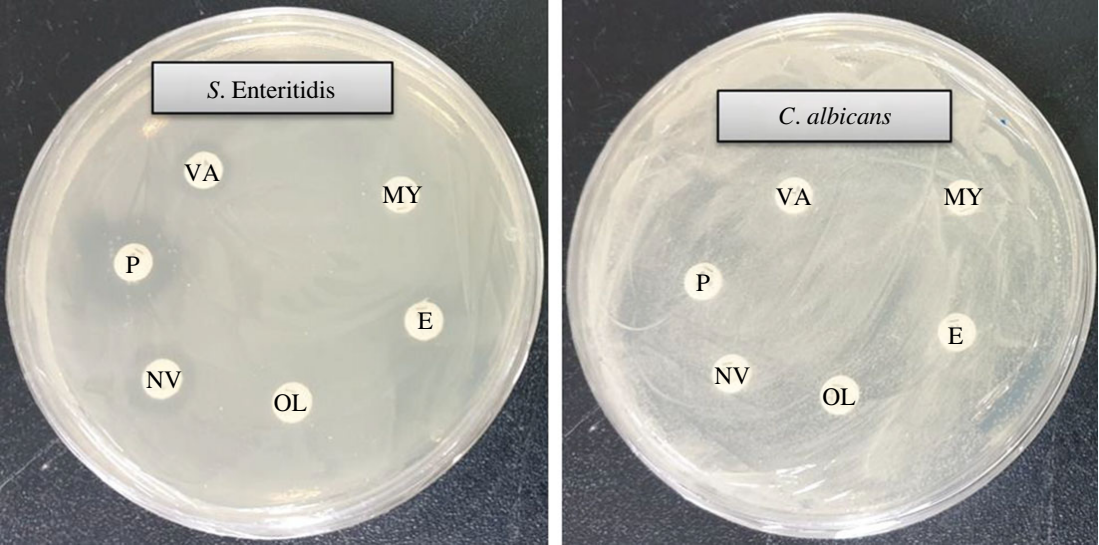
ZOI of tested antibiotics against *S.* Enteritidis and *C. albicans*. P, penicillin G; E, erythromycin; NV, novobiocin; OL, oleandomycin; VA, vancomycin; and MY, lincomycin.

In this study, the efficacy of six commercial antibiotics (novobiocin, penicillin G, erythromycin, oleandomycin, vancomycin and lincomycin) against *S.* Enteritidis and *C. albicans* was compared with the biosynthesized AgNPs (table 3). The results showed that all commercial antibiotics tested were ineffective against *C. albicans*, while novobiocin, penicillin G and vancomycin exhibited weak activity against *S.* Enteritidis when compared with the biosynthesized AgNPs. Figure 9 shows the results of the antibacterial activity of the commercial antibiotics. This finding is in line with previous studies that demonstrated the effectiveness of biosynthesized AgNPs as antimicrobial agents [18,19]. The antibacterial activities of the biosynthesized AgNPs indicate their potential as novel therapeutic agents against MDR pathogenic microorganisms.

**Table 3.** Antimicrobial impact of tested antibiotics against *S.* Enteritidis and *C. albicans*. —, no ZOI.

pathogenic species	antibiotic	ZOI (mm)
*Salmonella* Enteritidis [ATCC 13076]	oleandomycin	—
	penicillin G	14.5 ± 1.1
	novobiocin	10.5 ± 1.2
	lincomycin	—
	vancomycin	9.5 ± 1.0
	erythromycin	—
*Candida albicans* [KACC 30071]	oleandomycin	—
	penicillin G	—
	novobiocin	—
	lincomycin	—
	vancomycin	—
	erythromycin	—

### 3.6. Minimum inhibitory concentration and minimum bactericidal concentration

**Figure 10.**
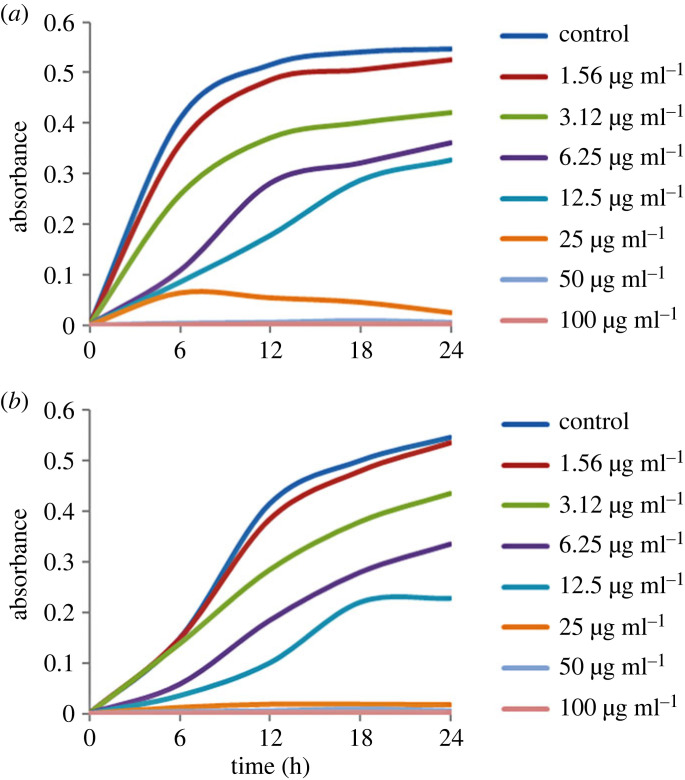
MICs of biosynthesized AgNPs against *S.* Enteritidis (*a*) and *C. albicans* (*b*).

To evaluate the MIC of AgNPs biosynthesized by *Paenibacillus* sp. MAHUQ-63 against *S*. Enteritidis and *C. albicans* different concentrations of the biosynthesized AgNPs were used. The results revealed that the AgNPs biosynthesized by *Paenibacillus* sp. MAHUQ-63 had a MIC of 12.5 and 25 µg ml^−1^ for *C. albicans* and *S*. Enteritidis, respectively. These findings demonstrate that the synthesized NPs effectively inhibited the growth of both *S.* Enteritidis and *C. albicans* (figure 10*a*,*b*). These MICs of the biosynthesized AgNPs were less than those of many other antibacterial and antifungal agents against *S.* Enteritidis and *C. albicans* [54,55].

**Figure 11.**
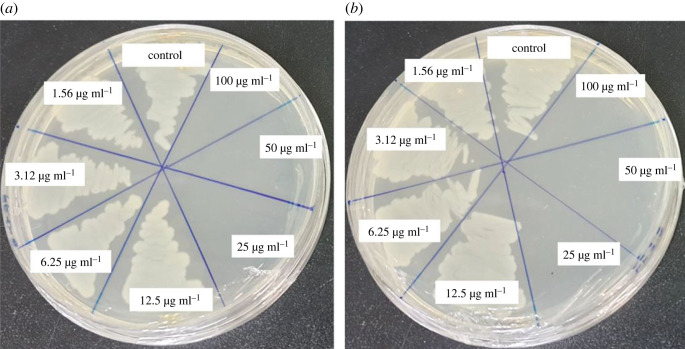
MBCs of biosynthesized AgNPs against *S.* Enteritidis (*a*) and *C. albicans* (*b*).

The MBC against both pathogenic *S.* Enteritidis and *C. albicans* was found to be 25 µg ml^−1^ (figure 11*a*,*b*). These results confirmed that the AgNPs biosynthesized by *Paenibacillus* sp. MAHUQ-63 efficiently inhibited the proliferation of both pathogenic *S.* Enteritidis and *C. albicans*.

### 3.7. Morphological evaluation

**Figure 12.**
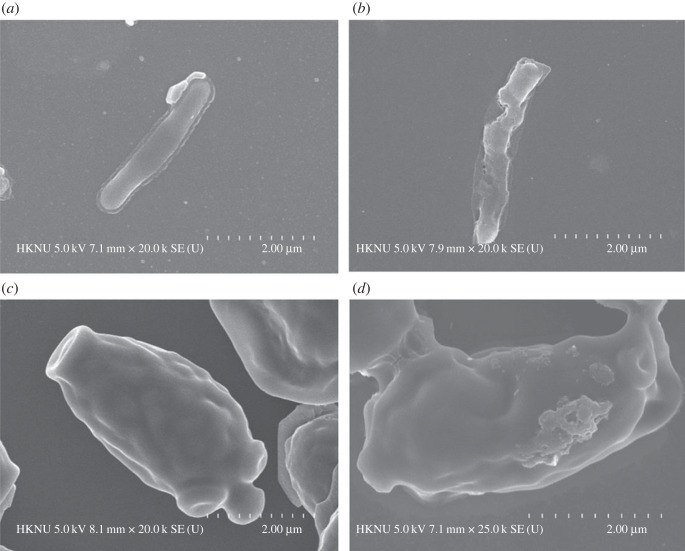
Normal *S.* Enteritidis (*a*), AgNPs-treated *S.* Enteritidis (*b*), normal *C. albicans* (*c*) and AgNPs-treated *C. albicans* (*d*).

The morphological changes induced by the *Paenibacillus* sp. MAHUQ-63-mediated biosynthesized AgNPs on the *S.* Enteritidis and *C. albicans* cells were investigated by FE-SEM (figure 12). The untreated *S.* Enteritidis cells exhibited normal rod-shaped morphology with an intact surface (figure 12*a*). However, treatment with 1 × MBC of biosynthesized AgNPs caused irregularities, damage and deformation on the outer surface of *S.* Enteritidis cells, leading to complete collapse of the cell membranes (figure 12*b*). Similarly, the untreated *C. albicans* cells displayed normal oval-shaped morphology with an intact surface (figure 12*c*). *Candida albicans* cells treated with AgNPs showed irregularities, damage and deformation on the outer surface (figure 12*d*). The structural changes and damage to the bacterial and fungal cell walls indicate that the *Paenibacillus* sp. MAHUQ-63-mediated synthesized AgNPs may disrupt normal cell functions and cause microbial cell death.

## 4. Discussion

The present study aimed to isolate and use a bacterial strain, *Paenibacillus* sp. MAHUQ-63, from a pumpkin garden, for the facile and rapid synthesis of AgNPs. The biosynthesis process involved the use of bacterial culture supernatant with silver nitrate solution. The optical properties of AgNPs were examined by UV–Vis spectroscopy based on their wavelength. The biosynthesized AgNPs exhibited an absorption peak at 440 nm, consistent with previously reported data of absorption peaks ranging between 400 and 500 nm [11,51]. The spherical shape of AgNPs was confirmed by FE-TEM analysis with 15–55 nm in size, similar to previous findings [19,23]. XRD analysis confirmed the crystallinity of the biosynthesized AgNPs, consistent with the literature on AgNPs biosynthesized using plants and microbes [13,52]. Furthermore, FTIR analysis indicated the involvement of different biomolecules in the reduction, capping and stabilizing of AgNPs.

**Figure 13.**
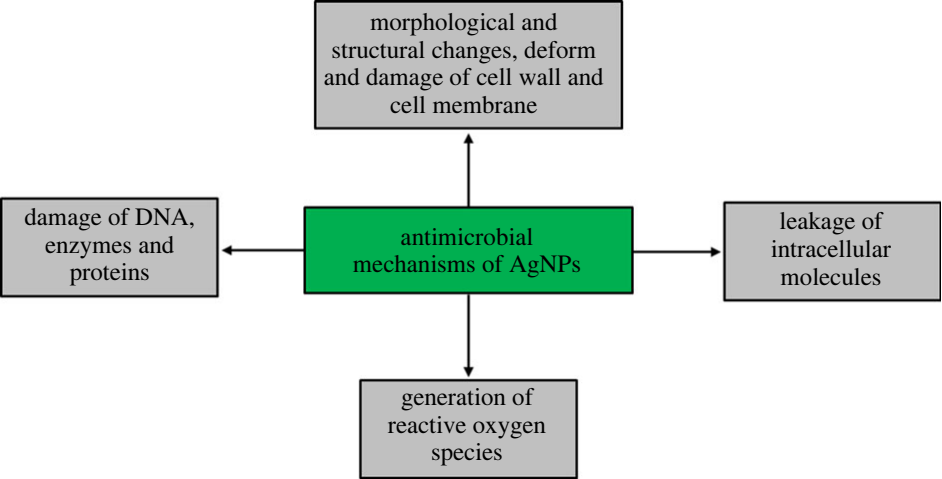
Schematic figure representing the proposed antimicrobial mechanisms of AgNPs.

The emergence of resistant microorganisms has limited the use of available antibiotics against microbial infections. Green synthesized AgNPs could be good candidates to solve this problem. There are several factors that may have an influence on the biological activity of synthesized NPs. These factors include size distribution, morphology, surface charge, surface chemistry, capping agents, etc. [56,57]. In this study, bioactive AgNPs were synthesized using the culture supernatant of *Paenibacillus* sp. MAHUQ-63. Green synthesized AgNPs showed remarkable antibacterial and antifungal activity against drug-resistant strains of *S.* Enteritidis and *C. albicans*. The ZOI diameters of synthesized AgNPs against *S.* Enteritidis and *C. albicans* were 18.0 ± 1.0 and 19.5 ± 1.3 mm, respectively, when treated with 100 µl of AgNPs solution at 500 ppm concentration. FE-SEM analysis showed that untreated *S.* Enteritidis and *C. albicans* cells had normal shapes and intact cell surfaces without any damage (figure 12*a*,*c*). However, after treatment with green synthesized AgNPs, both *S.* Enteritidis and *C. albicans* cells exhibited irregular, damaged and wrinkled surfaces (figure 12*b*,*d*). The structural changes and damage of bacterial and fungal cell walls indicated that AgNPs synthesized by *Paenibacillus* sp. MAHUQ-63 might disrupt normal cell functions and cause the death of microbial cells. AgNPs may also enter into the microbial cell and bind to DNA, which affects normal gene expression and metabolism [58–61]. Moreover, AgNPs may induce the formation of free radicals in microbial cells, leading to the damage of cell membranes [62,63]. AgNPs also may cause the leakage of intracellular molecules, and the denaturation of enzymes and proteins, and finally lead to the death of microbial cells [16,62,63]. Figure 13 shows the schematic illustration of the proposed antimicrobial mechanisms of AgNPs.

## 5. Conclusion

This is the first study to show the excellent capability of the culture supernatant of *Paenibacillus* sp. MAHUQ-63 as a reducing, capping and stabilizing agent for the green and efficient synthesis of bioactive AgNPs. The biosynthesized AgNPs were spherical and had sizes ranging from 15 to 55 nm. They were also stabilized by different functional groups on their surface. Synthesized AgNPs exhibited potent antibacterial and antifungal activity against drug-resistant *S.* Enteritidis and *C. albicans*. They also caused structural damage to the microbial cell walls and membranes. From the present findings, it can be concluded that AgNPs synthesized using the culture supernatant of *Paenibacillus* sp. MAHUQ-63 can be used as a novel antimicrobial agent to control antibiotic-resistant microorganisms, especially for treating *S.* Enteritidis and *C. albicans* infections.

## Data Availability

The data are provided in the electronic supplementary material [64].
